# Mechanistic Parameterization of the Kinomic Signal in Peptide Arrays

**DOI:** 10.4172/jpb.1000401

**Published:** 2016-05-24

**Authors:** Alex Dussaq, Joshua C Anderson, Christopher D Willey, Jonas S Almeida

**Affiliations:** 1 University of Alabama at Birmingham, USA; 2 Biomedical Informatics Department, Stony Brook University, USA

**Keywords:** Kinomics, JavaScript, Non-linear regression, Kinomic peptide array, PamGene

## Abstract

Kinases play a role in every cellular process involved in tumorigenesis ranging from proliferation, migration, and protein synthesis to DNA repair. While genetic sequencing has identified most kinases in the human genome, it does not describe the ‘kinome’ at the level of activity of kinases against their substrate targets. An attempt to address that limitation and give researchers a more direct view of cellular kinase activity is found in the PamGene PamChip® system, which records and compares the phosphorylation of 144 tyrosine or serine/threonine peptides as they are phosphorylated by cellular kinases. Accordingly, the kinetics of this time dependent kinomic signal needs to be well understood in order to transduce a parameter set into an accurate and meaningful mathematical model.

Here we report the analysis and mathematical modeling of kinomic time series, which achieves a more accurate description of the accumulation of phosphorylated product than the current model, which assumes first order enzyme-substrate kinetics. Reproducibility of the proposed solution was of particular attention. Specifically, the non-linear parameterization procedure is delivered as a public open source web application where kinomic time series can be accurately decomposed into the model’s two parameter values measuring phosphorylation rate and capacity. The ability to deliver model parameterization entirely as a client side web application is an important result on its own given increasing scientific preoccupation with reproducibility. There is also no need for a potentially transitory and opaque server-side component maintained by the authors, nor of exchanging potentially sensitive data as part of the model parameterization process since the code is transferred to the browser client where it can be inspected and executed.

## Introduction

Kinases have been extensively studied since the discovery of enzyme regulation via phosphorylation in the 1950’s. They represent more than 500 proteins and 100,000 phosphorylation sites [1]. They have been examined, among other things, as regulators, signal transducers, and are the second most drugged gene class [2]. While genetic sequencing has identified most kinases in the human genome, it does not describe the ‘kinome’, at the level of the activity of kinases on kinase targets. Kinases play a very important role in cancer development and kinomics, a global description of kinases and their substrates, shows great promise in the field of personalized medicine, correspondingly, numerous technologies have been developed to measure the kinome activity.

Of the techniques for kinome examination, peptide chips show significant promise due to several key features: 1) They can be used for high-throughput screening, 2) they allow the investigator to directly measure the effects of a drug, 3) they are comparably easy to create, and 4) they maintain similar enzyme kinetics to *in vivo* [3]. In particular, we are examining the PamGene PamChip© array, which allows a researcher to record and compare the phosphorylation of 144 13 amino acid long peptides containing one or more phosphorylatable residues [4].

**Figure 1:**
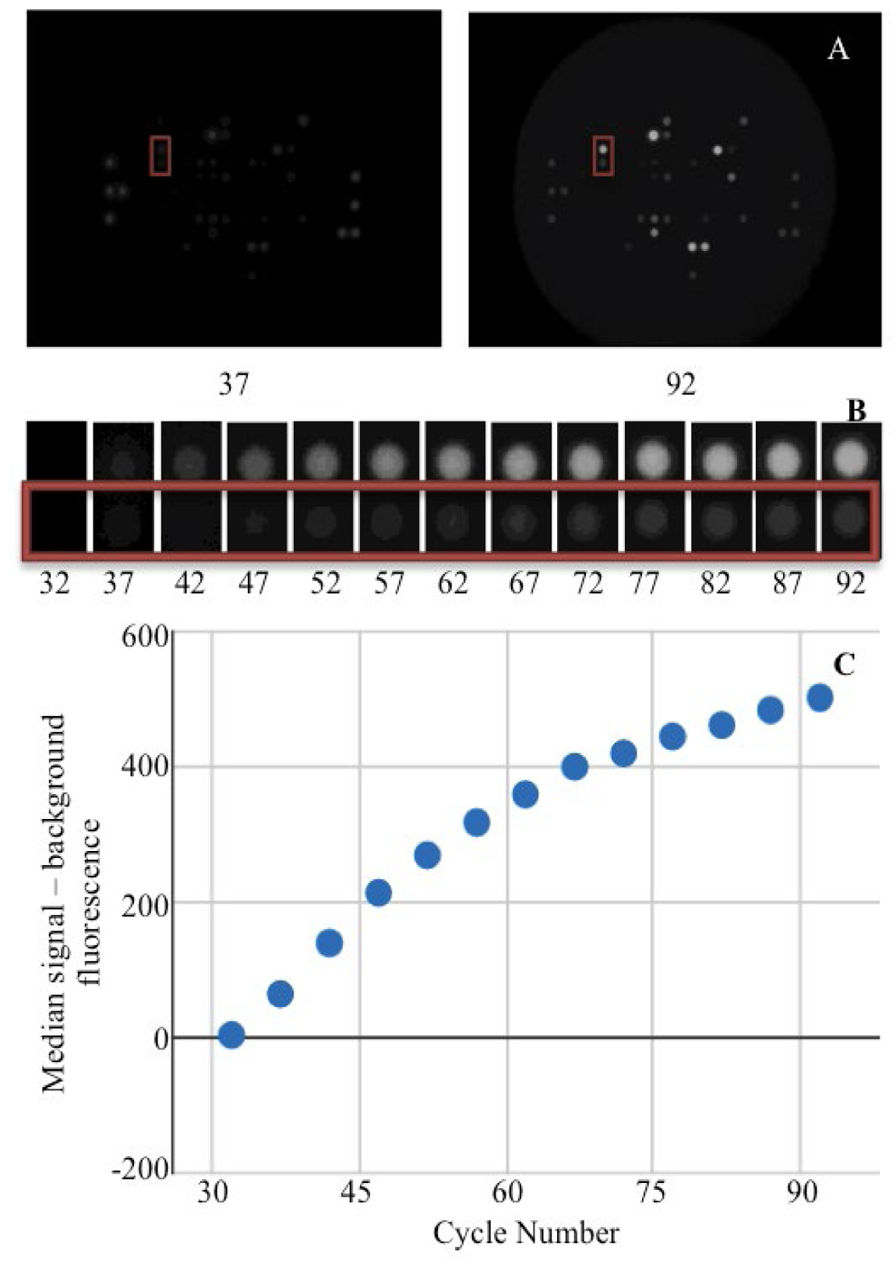
Representation of the data flow for the phosphorylation reaction for PamGene PamChip® experiments. Cycle number represents reaction time. Fluorescence intensity of peptide phosphorylation is recorded using a camera with a 50 ms exposure. All images were adjusted using color correction curve in Gimp (smooth curve x:225>10, y:225>165). (A) Two images taken during the phosphorylation reaction. (B) Two selected spots from (A) displayed for every time point. (C) Graphical representation of the median signal - background value calculated by the PamGene BioNavigator and plotted utilizing Google Charts.

Total protein lysates are prepared with protease and phosphatase inhibitors and 1–10 μg of lysate are mixed in kinase buffer with ATP and Mg^2+^. Samples are then loaded onto the PamStation© along with fluorescently labeled anti-phosphoserine, anti-phosphothreonine, or anti-phosphotyrosine antibodies. Using microfluidics, the sample is repeatedly pumped through an aluminum oxide matrix containing an array of phosphorylatable peptide probes. Active kinases within the lysate sample can phosphorylate these peptide probes that are then quantified by measuring the fluorescence of the phospho-specific antibodies using a charge-coupled device (CCD) camera. Each PamChip^®^ experiment produces two sets of data for 144 phosphorylatable amino acid residues. The first, a non-linear model, uses a camera exposure time of 50 ms to compare the phosphorylation of the residues at time points throughout the experiment (Figure 1). The second, a linear model, uses varying camera exposure time from 5–150 ms to quantify the end level phosphorylation following the washing away of the reactants. For the purposes of this publication we will discuss the first, non-linear, time series model.

Due to the nature of kinases, any of the 144 peptides will likely have numerous kinases acting upon them [5,6]; additionally the secondary step of antibody binding to produce a fluorescent signal further complicates the picture. This leads to a serious problem with deconvoluting the signal to the representative original kinases. In the literature upstream kinases are predicted using probable upstream kinase prediction linked back to biological pathways. This has shown utility in a wide range of disease models such as schizophrenia [7,8], HIV latency [9], renal cell carcinoma [10], Glioblastoma [11,12] and lung cancer [13]. Additionally a number of studies have utilized *ex vivo* treatments with kinase inhibitors not only against cells prior to lysis, but also treating lysates directly before profiling. In rectal cancer *ex vivo* treatment with the kinase inhibitor sunitinib was used to properly group and identify patients that would respond to both chemotherapy and radiotherapy [14] as well as to predict tumor cell dissemination within patients [15]. However, kinetic data, using a derivative of the curve fit at an early point (initial velocity or slope), was used in a similar drug study by Versele et al. [16] to comparatively measure, and predict response in 27 cancer cells to a multitargeted kinase inhibitor, a finding that was validated in xenograft tumor bearing mice. It is of the utmost importance that the signals created properly represent the data and introduce minimal error. Therefore we sought to investigate the error introduced by the time series curve fitting procedure.

The current model of the kinomic time series [17] relies on an exponential model, which is typically associated with processes as diverse as biological growth, radioactive decay, and first order enzyme kinetics, however given the complicated picture we present above we theorize that first order kinetics will not be the optimal method to represent the data.

(1) $$y=y_{0}+y_{\max}\cdot (1-e^{-c\cdot x})$$

As the results described below will confirm, we hypothesize, the biochemical processes underlying the production of the phosphorylated fluorescent signals are better described by hyperbolic, not exponential, activity curves.

It should be noted that previous studies by other authors have approached the analysis of the kinomic signal describing it with penalized smoothing splines [18]. However, like other smoothing approaches to signal description [19,20]; those approaches seek to subtract the statistical structure of signal noise rather than capturing the underlying mechanism. Since our goal is to translate the dynamics of the kinomic signal into a vector of parameters that can be mapped to biochemical mechanisms that line of work was not pursued here. In the same vein, this study does not approach the systems dynamics that multiple kinomic signals may in fact be describing collectively. That systems-level modeling of the kinase signal is approached in studies like “Mathematical Models of Protein Kinase Signal Transduction” [21]. More broadly, the description of multi-signal systems requires the adoption of more generic frameworks, if for no other reason than for the sake of maintaining parametric sensitivity. An excellent review of the generalization of individual reaction kinetics into broader Biochemical Systems Theory frameworks such as S-Systems can be found in Voit EO [22]. The relevant context for the study reported here is that parameterization of kinomic signals produces more a meaningful and accurate description of both individual signal and system-level kinase activity.

## Methods

### PamGene

PamGene utilizes a peptide array consisting of 144 unique peptides in approximately equal concentrations. Each peptide contains one or more phosphorylatable residues, and all peptides are simultaneously exposed to cell lysate containing active kinases. A detailed description of the sample preparation, processing, and analysis can be found here [11]. Once a residue is phosphorylated fluorescent antibodies bind the phosphorylated peptide residue. The signal recorded is then the amount of phosphorylation based on this fluorescence, for a series of time points [23]. The signal is normalized using PamGene^®^’s PAMCHIP EVOLVE software as part of the BioNavigator software suite in two major steps: (1) Image analysis segments the image to identify the spots and the local region around them, and then (2) local background pixels are identified as the corners of a square cell around the spots. Median signal over the spot is subtracted from the median background signal to produce a normalized value [24].

### Curve fitting

The numerical methods developed for this study sought to satisfy recommendations for reproducibility to the fullest. Accordingly, criteria were set to utilize an open source, version controlled, and web executable application. Those requirements were met by developing a JavaScript library implementing a simplified, portable, steepest-descent non-linear regression algorithm. Source code can be found (https://github.com/adussaq/amd_cf).

To avoid blocking the accompanying web application, this library was written to be run within web workers, using background processes supported by modern Web Browsers [25]. A specialized library that coordinates the web workers in the execution of this algorithm by queuing the asyncronous tasks was developed and is also made publicly available with open source at https://github.com/adussaq/amd_ww/. Both of these modules are designed to work with all modern web browsers including Mozilla’s Firefox, Google Chrome, and Apple’s Safari, both on the mobile and desktop platforms.

The iterative process to minimize the sum of square deviations is summarized below:

$$\begin{array}{l} X=[X_{1},\ldots X_{n}],\;X_{1}=[x_{1,1},\ldots ,x_{1,p}],\;Y=[y_{1},\ldots ,y_{n}],\;Y=f(X,P_{o})\\ P_{0}=[p_{0,1},\ldots ,p_{0,m}],\;P_{1}=[p_{1,1},\ldots ,p_{1,m}],\;S=[s_{1},\ldots ,s_{m}]\\ \mathrm{sse}(Y,f(X,P))=\sum_{q=1}^{n}{\left(f(X_{q},P)-Y_{q}\right)}^{2} \end{array}$$

1. *p_1,1_* = *s_1_* + *P_0,0_*
2. if *sse*(*Y*,*f*(*X*,*P_1_*))< *sse*(*Y*,*f*(*X*,*P_0_*))
  *P_0,1_* = *P_1,1_, s_1_* = *s_1_* * 1.2
  else
  *P_1,1_* = *P_1,0_, s_1_* = *s_1_* * −*0.5*
3. Repeat 1 – 2 for *p*_0,2_ through *p*_0,m_
4. Repeat 1 – 3 until end condition are satisfied

Where *P* is the constant parameter vector, *X* is an independent matrix; *Y* is the corresponding dependent variable for the equation *ŷ_i_=f(X,P). S* are the steps taken in each iteration of the algorithm and *sse* is the sum of square deviations. Several parameters may be set, including a max iteration count (default 1000), initial step (default 1/100 of *P*_0_), and minimum percent change of sum of square deviations (default 6e–5%). In addition to the parameter vector, this returns an R^2^ and a Wald-Wolfowitz (WW) Runs Test to measure goodness of fit. Initial parameters for each equation were determined by iteratively resolving the equation for individual points algebraically, as illustrated for equation 3 by the implementation of the method *setInitial* at http://bit.ly/1Yosos6. All initial parameterization procedures, are kept alongside the version controlled in the public github repository https://github.com/kinome.

**Figure 2:**
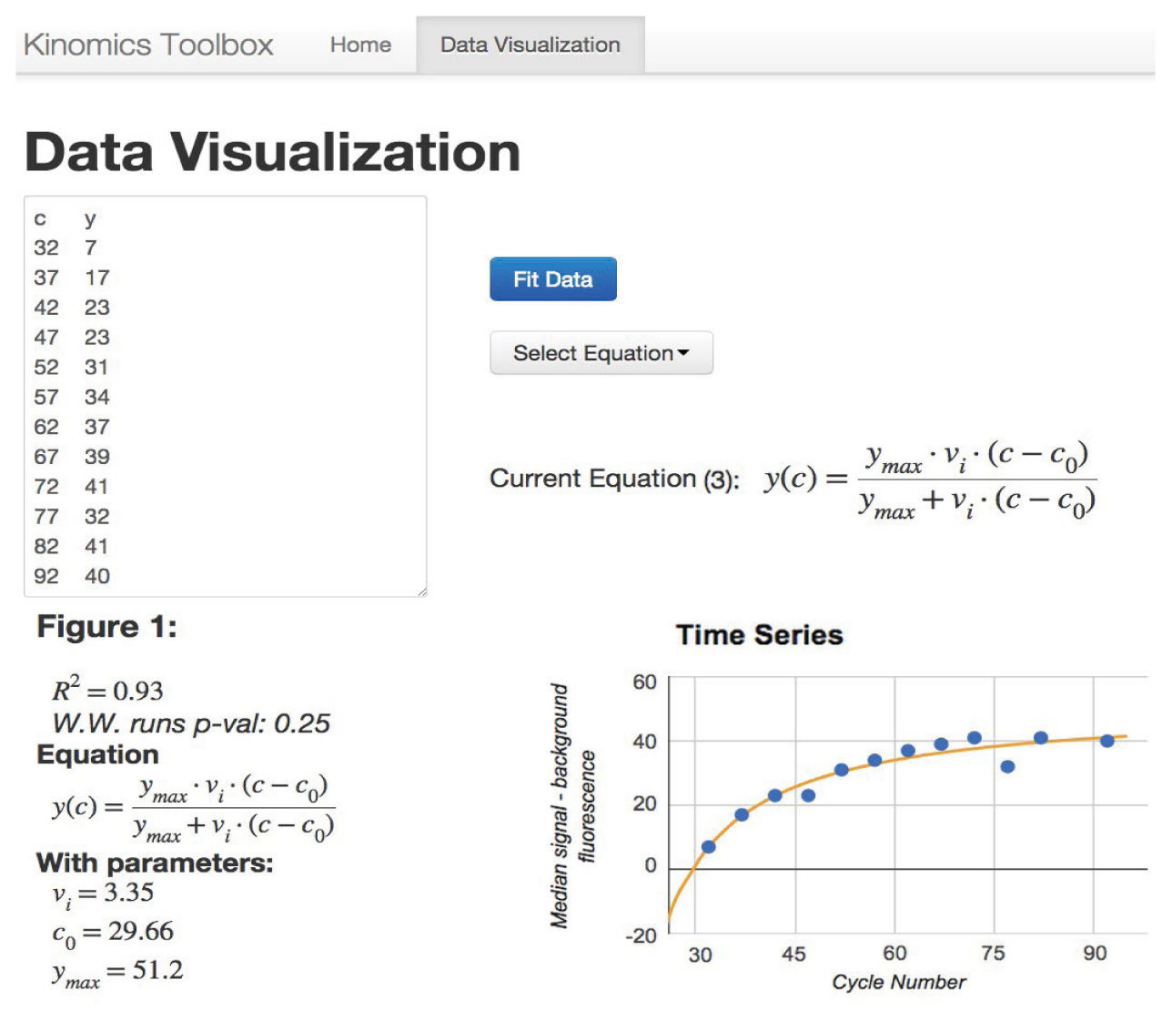
Screenshot of http://kinome.github.io/demo-cf/#model. The pictured tool is able to compare the three models utilizing any sample data. The top left is the editable data, the top right is the equation selection tool, and the bottom is the data and curve fit. Data is interactive, data points can be removed by clicking them, and their x, y-values are displayed on mouse over.

These modules were combined with the following visualization libraries: Google chart tools [26], jqmath [27], bootstrap [28] and jQuery [29] to create a tool to visualize individual curve fits. This is available at http://bit.ly/kinomic and shown in Figure 2. This represents a small example of these tools as they were applied to the remainder of the data.

### Model identification

Thirty-six samples ran utilizing protein tyrosine kinase (PTK) chips were selected to represent the global analytical space. These samples were selected to cover the spectrum of good to bad signal observations. These kinomic experiments included lysates derived from short-term frozen primary human tumor tissue, long term frozen primary human tumor tissue, and freshly lysed cultured human tumor and non-tumor cells. Lysates treated both *in vitro*, and *ex vivo* (on chip) with kinase inhibitors were included. More information on samples selected can be found in Table S1.

### Quality control

Since poor data fitting can be due to a number of problems, we selected only the data sets that had a Wald-Wolfowitz (WW) runs test with a p-value ≥ 0.05 for all three models. This choice was based on experimentation comparing this selection method to R^2^ (Figure S1). This reference data was converted into density scores using the Matlab package kde [30] (available at: http://bit.ly/kde_botev).

### Technical replicates

Two sets of 6 technical replicates for PTK data that passed QC as described above individually for models 2 and 3 (results) were selected for analysis. The values were exported, and the reproducibility of key parameters along with the calculated v_ini_ for model 2 (results) were investigated using transformed non-parametric quantile data for each set of replicates. This data was then converted to density measurements using Kernal Density Estimation [31] with *Parzen–Rosenblatt rule of thumb window*.

### Results

The data analysis that produced the results described in this section can be reproduced for arbitrary datasets by using the web application at http://bit.ly/kinomic, depicted in Figure 2. As detailed in the methods, no data is transferred out of the user’s web browser - all computation happens in the browser, and the code can be inspected by opening the developer tools or by utilizing the github repository (for example, see https://developer.chrome.com/devtools for Google Chrome, or https://developer.mozilla.org/en-US/docs/Tools for Mozilla Firefox).

### Model identification

The PamGene kinase peptide arrays generate two unique sets of fluorescence intensity data for each sample run: a post-wash linear series and a nonlinear time progression. The post-wash linear data is obtained by varying the camera exposure time following washing off of remaining sample and reagents. The time series data however, is obtained as the reaction progresses at set intervals based on the number of cycles the machine has run. Three models were investigated: a simple negative exponential (equation 1), a background corrected (c_0_) variation of the negative exponential (equation 2) and a background corrected (c_0_) rational hyperbolic (equation 3).

(1) $$y=y_{0}+y_{\max}\cdot (1-e^{-k\cdot c})$$

(2) $$y=y_{0}+y_{\max}\times (1-e^{-k\cdot (c-c_{0})})$$

(3) $$y=\frac{y_{\max}\cdot v_{i}\cdot (c-c_{0})}{y_{\max}+v_{i}\cdot (c-c_{0})}$$

In each of these models, *y* represents the amount of phosphorylation measured as median signal minus the background, at cycle number, *c*. The value y_max_ is the upper asymptotic value y can reach, whereas v_i_ is initial slope of y and k represents the rate of exponential growth. Equation 3 was derived as follows:

$$\begin{array}{l} y=v_{i}\cdot c\;[\text{Linear}\;\text{accumulation}\;\text{of}\;a\;\text{phosphorylated}\;\text{product}\;c:dy/dc=v_{i}]\\ y=v_{i}\cdot c(1-y/y_{\max})\\ \left[\text{Limited}\right]\\ y=v_{i}c-\frac{v_{i}cy}{y_{\max}}↔y\left(1+\frac{v_{i}c}{y_{\max}}\right)=v_{i}c↔\\ ↔y={\left({\left(v_{i}c\right)}^{-1}+{y_{\max}}^{-1}\right)}^{-1}↔\\ ↔y=\frac{y_{\max}\cdot v_{i}\cdot (c-c_{0})}{y_{\max}+v_{i}\cdot (c-c_{0})} \end{array}$$

Due to the rate of asymptotic approarch being higher in an exponential equations than in a rational ones all data will produce different y_max_ predictions based on whether model 1/2 or model 3 is utilized. Since this is a predictive term and by definition never truly reached it cannot be expected to be accurate and should not be used for comparisons.

### Model identification

**Figure 3:**
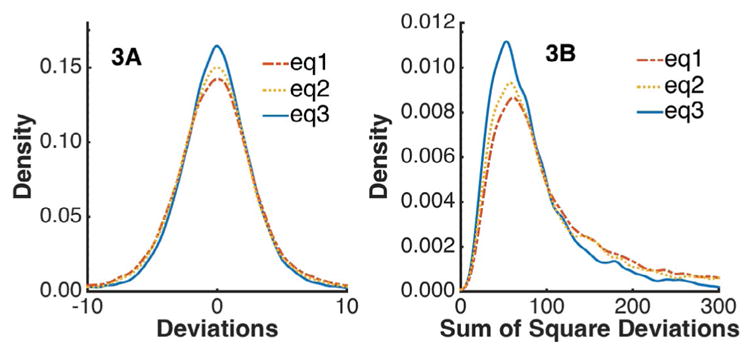
Graphical representation of residual variation in background fluorescence versus cycle (see Figure 2) for the three models. *eq3* can be seen as the solid blue line in both panels of the figure as the highest peak. *eq2*, and *eq1* peak lower respectively and can be seen to follow *eq3* as a dotted and a slash dot line in both panels. These lines were generated by fitting 36 PTK experiments (see Methods) to all three models. Following quality control 2863 fits remained across all three models; deviations were calculated and plotted for density.

The thirty-six samples analyzed to represent the global analytical space generated 5184 time series (36 series x 144 kinases). Each of them was parameterized for each of the 3 equations. Of those, 2863 (55%) had a Run’s test p-value ≥ 0.05 across all three models, (eq1 2924, eq2 3142, eq3 3342) passing quality control (see Methods). Figure 3a shows the distribution of residuals across this sample data (37,219 points x 3 models). Peak densities were as follows, eq1-0.142, eq2-0.150, eq3-0.165. This suggests that model 3 offers a better description of the kinomic signal (See Discussion). That superiority is reinforced by the distribution of the sum of square deviations for each fit (2863 points x 3 models) depicted in Figure 3b, where equation 3 is observed to lead to narrower residual distributions. Peak residual densities occurred at the following points: eq1- (60.7, 0.00866); eq2- (56.8, 0.00932); eq3- (52.8, 0.0112). While the differences presented are minor, it is important to note that equation 3 improves upon equation 2 while reducing the number of parameters from 4 to 3.

### Reproducibility

The variability of the kinomic signal was assessed by analyzing two sets of 6 technical replicates. Based on the above results models 2 and 3 were investigated for the reproducibility of their key parameters. Each model was filtered individually by QC (methods), and then v_ini_, the value typically utilized in current publications, was calculated for model 2 as follows:

$$v_{\mathrm{ini}}=y^{\prime }(35)=y_{\max}\cdot k\cdot e^{-k\cdot (35-c_{0})}$$

**Figure 4:**
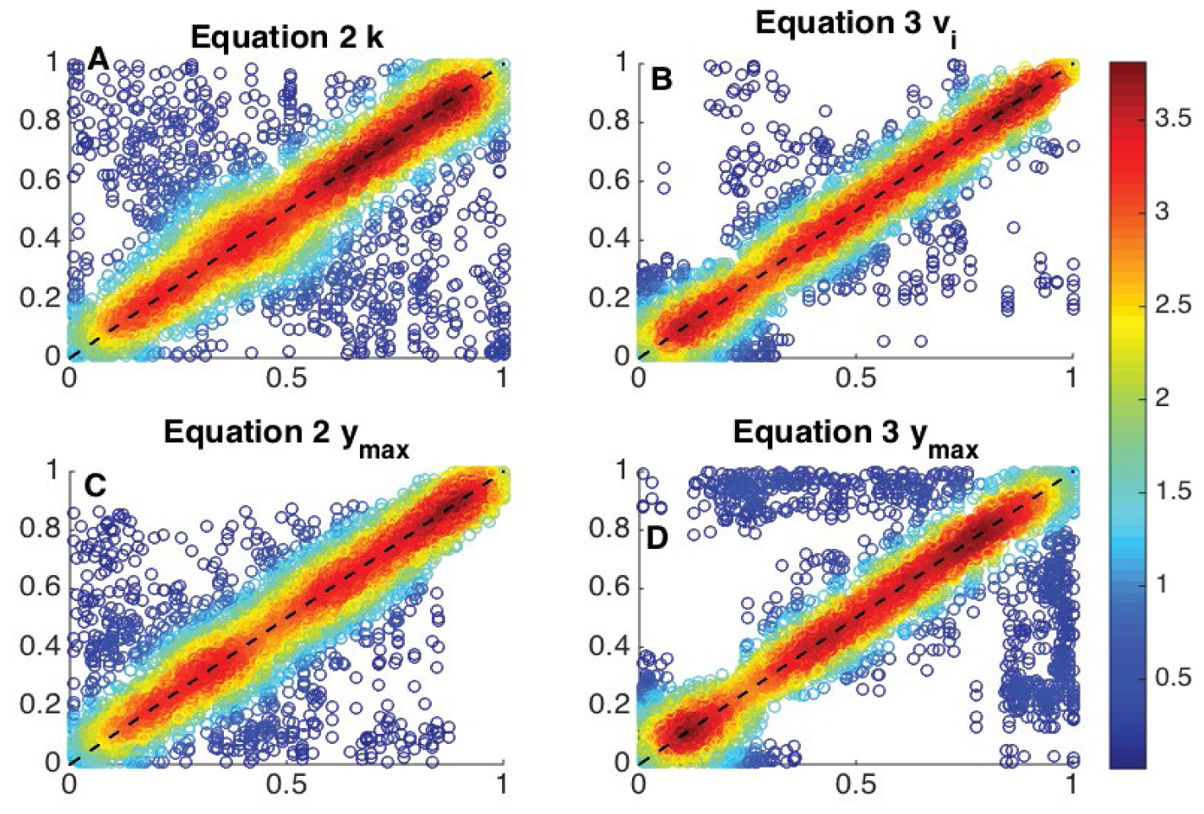
Analysis of reproducibility of parameterizations in two 6 replicate sets. Ideally all data sits on the dashed 1-1 line. Color represents relative density with red being the highest. Panel A is the key parameter k for equation 2 (results) and has a Spearman’s rank correlation of 0.7101. Panel B is the key parameter v_i_ for equation 3 (results) and has a Spearman’s rank correlation of 0.9352. Panel C is the predictive parameter y_max_ for equation 2 (results) and has a Spearman’s rank correlation of 0.8569. Panel D is the predictive parameter y_max_ for equation 3 and has a Spearman’s rank correlation of 0.7751. These were produced across 144 peptides, with 12 technical replicates, after filtering for high quality fits (See Methods) this created 5306 points (of a possible 8640) for panels A/C and 6802 (of a possible 8640) for panels B/D. k/v_i_ are the critical values for defining the curves.

These values were non-parametrically pre-processed by replacing raw values by the corresponding quantiles, for *k*, v*_ini_* and y*_max_* from model 2 and v*_i_* and y*_max_* from model 3. The combined sets of technical replicates had the following quantile-quantile correlations: *cor(q – q: equation 2, k) =0.7101; cor (q – q: equation 2, y_max_) =0.8569; cor (q* − *q: equation 2, v_ini_) =0.9359* (not pictured)*; cor (q – q: equation 3, v_i_) =0.9352, cor (q* − *q: equation 3, y_max_) =0.7751.* Equation 2 had a total of 1252 (of 1728) successful fits and Equation 3 had a total of 1503 (of 1728) successful fits. The results obtained are presented in Figure 4. These results indicate y*_max_* is more reproducible for model 2 than model 3. However the stability of this parameter is low for both models. This instability is due to the large number of curves produced remaining in a near linear or linear phase. This results in y*_max_* values that far exceed the boundaries of the data presented and reinforces the idea that y*_max_* should not be utilized for comparative analysis. The reproducibility of the key parameter for equation 3 (v*_i_*) is a substantial improvement over the key parameter for equation 2 (k). Interestingly, the reproducibility of v*_ini_* as calculated from equation 2 is a slight improvement (Correlation difference of 0.005) over that of the v*_i_* from equation 3. More importantly, v*_ini_* is more reproducible than the parameters utilized to calculate it. This indicates that the parametric stability for equation 2 is low even though the estimation early slope is stable.

## Discussion

The kinome represents a very functional subset of the genome and is of high interest to academia and pharma. Kinases are highly druggable and kinase targeted agents have generated very promising results in the clinic, particularly in proliferative diseases. However, the enzymatic nature and “promiscuity” of kinases produces significant challenges to studying the kinome. Indeed, an individual kinase typically targets multiple substrates with varying affinities while substrates are often targeted by more than one kinase. Enzymatically, this can be seen in the non-linear curves generated by the time series PamStation data. The velocity of the reaction and shape of the curve will vary based on the kinase (or kinase family) and substrate affinities. Biologically, this manifests as molecular redundancy where multiple signals can converge on the same target protein.

Of the three models investigated the three-parameter rational model for eq3 most closely described the data for the following reasons:

1. Having the highest peak for the deviation plot and for the model sum of square deviations (Figure 3).
2. Having the highest success rate using the curve fitting algorithm, 64.5% (+3.9% over *eq2*, mixed quality data), and 87.0% (+14.5% over eq2, high quality data) as determined by WW runs test (Methods). This indicates a higher randomness in the non-parametric distribution of residuals for eq3.
3. Utilizing only three parameters to describe the model. Based on the above metrics, the four-parameter exponential model (eq2) is the second best option; however the higher number of parameters reinforces the use of eq3.
4. Having the key parameter with the highest reproducibility. The reproducibility of the key parameter, vi from model 3 is significantly higher than that of k from model 2 (+31.7%).
5. Qualitatively, rational models are commonly used in enzyme kinetics with one limiting reaction, while exponential models are utilized for first order reaction with one reagent.

Generally adding parameters to a model decreases the error in residuals by allowing additional variability to be accounted for. However when moving from the four-parameter eq2 to the three-parameter eq3, we do not see worsening of residuals, nor a decrease in reproducibility. In fact we observe the opposite. This decrease in parameter space becomes particularly important when considering individual fits contains only 13 points. This move then represents an 11% increase in the degrees of freedom with an overall improvement in multiple goodness-of-fit metrics. It is important to note, previously published kinetic studies focus on the calculated value: v*_ini_*. Our analysis indicates, when calculated from eq2, the reproducibility of v*_ini_* is nearly equivalent to that of the comparable parameter, v*_i_* from eq3. Once again, eq3’s use of one less parameter to produce equivalent or improved parameterization solidifies the suggested use of eq3.

This is the first time, to the authors’ knowledge, that even a preliminary assessment of the variability and reproducibility of the PamGene PamChip^®^ has been investigated in the literature. Since future goals include tailoring cancer treatments to patients, a thorough investigation of this nature is necessary. In addition to the reproducibility of the data itself, the reproducibility and accuracy of the analysis itself is equally important. To this end all data analysis procedures were coded as web applications - that is, they are available as JavaScript scripts, in the open source and versioned environment of GitHub (see results and methods). The relevant feature of this approach, which we have explored and discussed in bioinformatics applications ranging from image analysis [32] to sequence analysis [33], is that the analysis can be repeated in any web browser, with easily open reviewable code, and without need to download nor install any components. In a nutshell, this creates lasting reproducibility that does not depend on server-side resources maintained by the authors of this report, nor requires any client-side configuration by users of the tools.

## Conclusion

Kinomic signals, as assessed by the PamGene platform, are accurately described as a rational hyperbolic function, not dissimilar in shape to Michaelis–Menten. This conclusion is presented here not as an alternative to noise filtering approaches followed by some software packages but, on the contrary, to inform the noise structure associated with the kinomics signal. Our original hypothesis that a non-exponential model would be provide a superior parameterization has been verified by an at least equivalent fit with a smaller parameter space, an essential characteristic given the limited data points per fit. A web application was developed and is made publicly available in an open source format to allow dissemination of libraries needed to parameterize the corresponding hyperbolic model. The reliance on the scripting language of the web, JavaScript, to develop those libraries, and depositing them in versioned GitHub pages is argued to maximize the reproducibility and reuse of the libraries developed. The main remaining challenge of the parameterization of kinomics signals appears to be associated with the robustness of the non-linear regression. Improvements in the parameterization procedure will decrease the number of individual kinomic signals that currently do not pass quality control.

## Supplementary Material

Suppl
