# Supplementary material for: Mechanistic Parameterization of the Kinomic Signal in Peptide Arrays
Source: J Proteomics Bioinform. Author manuscript; Available in PMC 2016 Sep 4. (PMC5010871; doi:10.4172/jpb.1000401)
Supplement: Suppl [file NIHMS808042-supplement-Suppl.pdf]

Barcode Array	Cell Description	Experimental Variable	Overall Signal/Curvefit Quality (High >70% of peptides fit on average across three models, Low < 50%. [fit defined as R^2 > 0.8])
631030521A1	Primary Tumor	basal	High
631030521A2	Primary Tumor	20nM erlotinib	High
631030521A3	Primary Tumor	500nM erlotinib	High
631030521A4	Primary Tumor	20uM erlotinib	High
631030522A1	Primary Tumor	basal	High
631030522A2	Primary Tumor	20nM crizotinib	High
631030522A3	Primary Tumor	500nM crizotinib	High
631030522A4	Primary Tumor	20uM crizotinib	Medium
631030523A1	Primary Tumor	basal	High
631030523A2	Primary Tumor	20nM lapatinib	High
631030523A3	Primary Tumor	500nM lapatinib	High
631030523A4	Primary Tumor	20uM lapatinib	High
631030701A1	Cultured Human tissue	Collection time 0	Low
631030701A2	Cultured Human tissue	Collection time 1	Low
631030701A3	Cultured Human tissue	Collection time 2	Medium
631030701A4	Cultured Human tissue	Collection time 3	Low
631030703A1	Cultured Human tissue	Collection time 0	Medium
631030703A2	Cultured Human tissue	Collection time 1	Low
631030703A3	Cultured Human tissue	Collection time 2	Medium
631030703A4	Cultured Human tissue	Collection time 3	Low
631030704A1	Cultured Human tissue	Collection time 0	Medium
631030704A2	Cultured Human tissue	Collection time 1	Low
631030704A3	Cultured Human tissue	Collection time 2	Medium
631030704A4	Cultured Human tissue	Collection time 3	Low
631207418A1	Primary Tumor	tumor1	Medium
631207418A2	Primary Tumor	tumor2	Medium
631207418A3	Primary Tumor	tumor3	Low
631207418A4	Primary Tumor	tumor4	Low
631207419A1	Primary Tumor	tumor5	Medium
631207419A2	Primary Tumor	tumor6	Low
631207419A3	Primary Tumor	tumor7	Medium
631207419A4	Primary Tumor	tumor8	Low
631207420A1	Primary Tumor	tumor9	Medium
631207420A2	Primary Tumor	tumor10	High
631207420A3	Primary Tumor	tumor11	Medium
631207420A4	Primary Tumor	tumor12	Low

Supplementary Table 1: List of the samples from which the global analysis was performed.

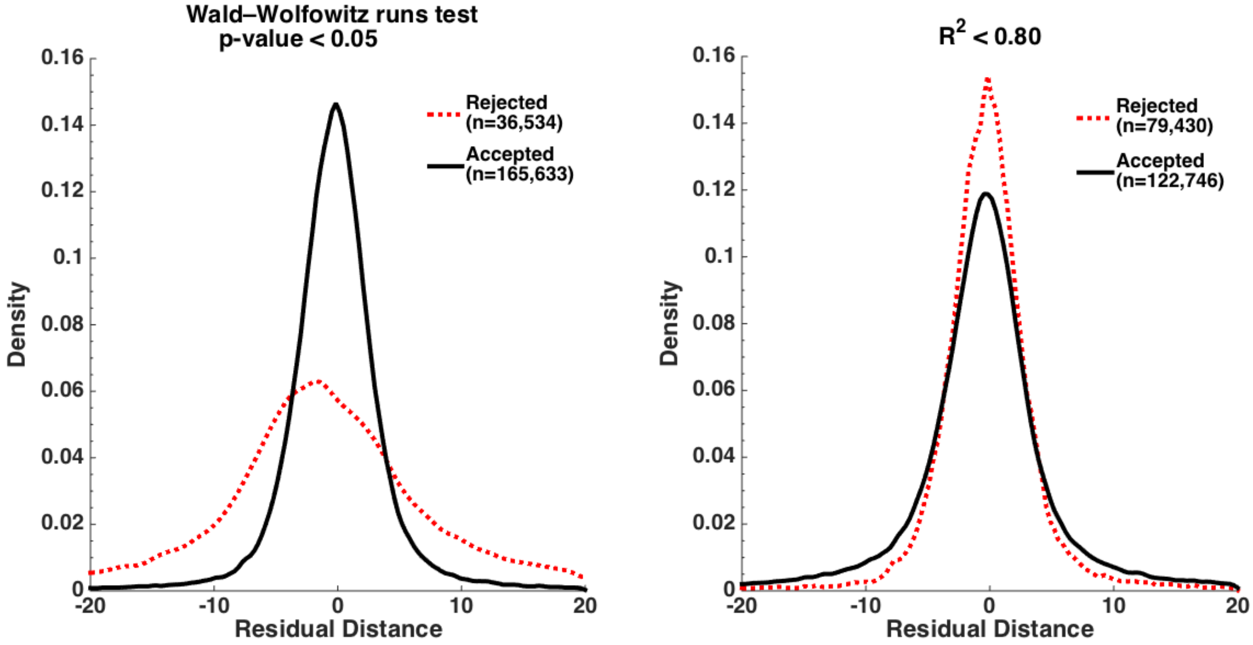

Supplementary Figure 1: Analysis of different methods of model filtering. Panel A shows the distribution of residual distance when selecting fits across all three purposed models for the 36 PTK experiments analyzed.
